# New records for *Anaplasma phagocytophilum* infection in small mammal species

**DOI:** 10.1186/s13071-018-2791-y

**Published:** 2018-03-20

**Authors:** Ioana Adriana Matei, Gianluca D’Amico, Angela Monica Ionică, Zsuzsa Kalmár, Alexandra Corduneanu, Attila D. Sándor, Nicodim Fiţ, Liviu Bogdan, Călin M. Gherman, Andrei Daniel Mihalca

**Affiliations:** 1 Department of Parasitology and Parasitic Diseases, Faculty of Veterinary Medicine, University of Agricultural Sciences and Veterinary Medicine, Cluj-Napoca, Romania; 2 Department of Microbiology, Immunology and Epidemiology, Faculty of Veterinary Medicine, University of Agricultural Sciences and Veterinary Medicine, Cluj-Napoca, Romania; 3 Department of Reproduction, Obstetrics and Pathology of Animal Reproduction, Faculty of Veterinary Medicine, University of Agricultural Sciences and Veterinary Medicine, Cluj-Napoca, Romania

**Keywords:** *Anaplasma phagocytophilum*, Small mammals, Prevalence, Romania

## Abstract

**Background:**

Tick-borne diseases pose a major threat in public health. The epidemiological dynamics of these diseases depends on the tick vector species and their hosts, as well as the geographical distribution and ecology of both. Among many possible hosts for ticks, small mammals have a major role in the development of immature stages of several tick species. Small mammals are also important reservoir hosts for several pathogenic agents and possible reservoirs for *Anaplasma phagocytophilum.* In this context, the aim of our study was to evaluate the prevalence of *A. phagocytophilum* in small mammal species in Romania.

**Results:**

A total of 791 small mammals of 31 species were tested by PCR, targeting the *rrs* gene for detection of *A. phagocytophilum* DNA. Positive results were obtained in 20 small mammals: five *Apodemus flavicollis* (6.49%), three *Sorex araneus* (9.09%), three *A. uralensis* (4.84%), two *A. sylvaticus* (3.92%), and one of each *Spermophilus cittelus* (7.14%), *Microtus agrestis* (3.85%), *Sorex minutus* (3.85%), *Muscardinus avellanarius* (3.13%), *Crocidura suaveolens* (2.44%), *Mus spicilegus* (2%) and *M. arvalis* (1.75%).

**Conclusions:**

Eleven small mammal species were found to be carriers of *A. phagocytophilum*, suggesting a possible involvement of these species in its epidemiology. To our knowledge, this is the first report of *A. phagocytophilum* in *S. minutus*, *C*. *suaveolens*, *M. spicilegus*, *M. avellanarius* and *S. citellus*.

**Electronic supplementary material:**

The online version of this article (10.1186/s13071-018-2791-y) contains supplementary material, which is available to authorized users.

## Background

Small mammals (Orders Rodentia and Eulipotyphla) represent a very diverse group of terrestrial vertebrates, with a worldwide distribution and usually represented by large populations [1, 2]. They are highly adapted for various types of habitat, including urbanized areas, being a link between wild and anthropomorphic ecosystems through the frequent movement of these animals and their ticks between human dwellings and natural environments [3]. Fluctuations in their densities are very important factors of disease risk [4], playing an important role in the ecology of ticks and tick-borne diseases.

Small mammals are important hosts for several tick species, having an essential role in the development of immature stages of hard ticks, and also being essential maintenance hosts for the immature stages of *Ixodes ricinus* [1, 5]. For instance, in a study focused on rodent-tick associations in Romania, a high prevalence (over 50%) of tick parasitism was found especially in *Microtus arvalis*, *Apodemus uralensis*, *Apodemus flavicollis* and *Myodes glareolus* [6]. Among the tick species found on small mammals in the Palaearctic, the genus *Ixodes* is the most well-represented: *I. angustus*, *I. apronophorus*, *I. crenulatus*, *I. hexagonus*, *I. laguri*, *I. nipponensis*, *I. occultus*, *I. pomerantzevi*, *I. redikorzevi*/*I. acuminatus*, *I. ricinus* and *I. trianguliceps* (reviewed in [1]). Even if some of these ticks are endophilic (nidicolous) (e.g. *I. trianguliceps* and *I. acuminatus*) and normally do not pose a direct public health hazard since they do not feed on humans, their co-occurrence with *I. ricinus* on the same host can lead to an exchange of pathogens among the different tick species [6]. From this point of view, small mammals are considered important bridge-hosts and pose an important risk for public health for numerous zoonotic pathogens [2]. Furthermore, rodents are often competent reservoirs for multi-host pathogens. For instance, mice (Muridae) and voles (Microtidae) are known to be important reservoirs for zoonotic agents like tick-borne encephalitis virus (TBEV), *Borrelia afzelii*, and “*Candidatus* Neoehrlichia mikurensis” [7]. The role of small mammals in the epidemiology of *A. phagocytophilum* in Europe is under debate, previously being considered important reservoir hosts [8]. Considering the high diversity and ubiquity of small mammals and the risk of human contact to their environment (nearly half of Romanian population live and work in rural areas and maintain close contact with nature [9]), the aim of this study was to evaluate the host and genetic diversity of *A. phagocytophilum* in small mammals in Romania.

## Methods

### Small mammals trapping and sampling sites

A total of 791 small mammals from 31 species were collected from a variety of habitats in 14 counties in Romania between 2010 and 2015, as previously described using snap traps [6]. The trapping of rodents was performed once per location in different months from late spring to early autumn when the vectors are active. In addition, other small mammals which were found dead, were collected from various sources. Whenever needed, research permits were obtained from competent authorities and ethical committees. Each captured or collected small mammal was identified to species level (according to [10]) and a necropsy was performed. Spleen tissue samples were collected from each animal. Ticks from the animals were removed and morphologically identified and published previously [6].

### DNA extraction

Genomic DNA extraction was performed from the spleen tissue using ISOLATE II Genomic DNA Kit (Bioline, London, UK), following the manufacturer’s instructions. For each extraction procedure, negative controls were used in order to identify possible cross-contamination. DNA from a representative number of samples was quantitatively analyzed using a Nanodrop ND-1000 spectrophotometer analyzer (Thermo Fisher Scientific Inc., Waltham, MA, USA).

### Polymerase chain reaction (PCR)

The presence of *A. phagocytophilum* DNA in the spleen tissue was tested by a series of nested PCR assays using specific primers amplifying fragments of *rrs* (1st PCR: ge3a/ge10r; 2nd PCR: ge9f/ge2) [11]. The amplification was performed as follows: 25 μl reaction mixture containing 12.5 μl of Green PCR Master Mix (Rovalab GmBH, Teltow, Germany), 6.5 μl PCR water, 1 μl of each primer (0.01 mM) and 4 μl aliquot of isolated DNA (1 μl of the primary PCR for the nPCR). The amplification profile for PCR consisted of 5 min of initial denaturation at 95 °C, followed by 40 cycles of denaturation at 94 °C for 30 s, annealing at 55 °C for 30 s and extension at 72 °C for 60 s, and a final extension at 72 °C for 5 min. For the nPCR, the amplification profile consisted of 5 min of initial denaturation at 95 °C, followed by 30 cycles of denaturation at 94 °C for 30 s, annealing at 55 °C for 30 s and extension at 72 °C for 60 s, and a final extension at 72 °C for 5 min. In each PCR reaction set (48 samples), one positive and two negative controls were included in order to assess the specificity of the reaction and the possible presence of the presence cross-contamination. Positive controls consisted of DNA extracted from a tick positive for *A. phagocytophilum* previously confirmed by sequencing [12] and negative controls consisted in sterile water. The PCR was carried out using a T100™ Thermal Cycler (Bio-Rad, London, UK).

### Agarose gel electrophoresis

PCR products were visualized by electrophoresis in a 1.5% agarose gel stained with SYBR® Safe DNA gel stain (Invitrogen, Carlsbad, CA, USA) and their molecular weight was assessed by comparison to a molecular marker (O’GeneRuler™ 100 bp DNA Ladder, Thermo Fisher Scientific Inc., Waltham, MA, USA).

### DNA sequencing

All positive PCR samples were sequenced. PCR products were purified from amplicons using the QIAquick PCR Purification Kit (Qiagen, Hilden, Germany). Sequencing analysis was performed (Macrogen Europe, Amsterdam, the Netherlands) and the obtained sequences were compared with those available in GenBank™ by Basic Local Alignments Tool (BLAST) analysis.

### Statistical analysis

Statistical analysis was performed using Epi Info™ 7 (CDC, Atlanta, GA, USA) software. The total infection prevalence of *A. phagocytophilum* (95% CI), the infection prevalence per species and group of species and the infection prevalence in each county was assessed using the Chi-square independence test. A *P*-value less than 0.05 was considered significant.

## Results

Twenty out of 791 small mammals were positive for *A. phagocytophilum* DNA presence with an overall prevalence of 2.53% (95% CI: 1.59–3.95%). The small mammal species found positive were: *S. araneus* (9.09%, 95% CI: 1.92–24.33%), *S. cittelus* (7.14%, 95% CI: 0.18–33.87%), *A. flavicollis* (6.49%, 95% CI: 2.14–14.51%), *A. uralensis* (4.84%, 95% CI: 1.01–13.5%), *A. sylvaticus* (3.92%, 95% CI: 0.48–13.46%), *M. agrestis* (3.85%, 95% CI: 0.10–19.64%), *S. minutus* (3.85%, 95% CI: 0.10–19.64%), *M. avellanarius* (3.13%, 95% CI: 0.08–16.22%), *C. suaveolens* (2.44%, 95% CI: 0.06–12.86%), *Mus spicilegus* (2%, 95% CI: 0.05–10.65%) and *M. arvalis* (1.75% ; 95% CI: 0.04–9.39%) (Table 1). No statistically significant differences in prevalence were observed between different small mammal species, nor between taxonomic groups: mice (3.06%, 95% CI: 1.61–5.56%), voles (1.14%, 95% CI: 0.14–4.07%), shrews (3.94%, 95% CI: 1.28–8.95%) dormice (1.33%, 95% CI: 0.03–7.21%), or squirrels (6.67%, 95% CI: 0.17–31.95%).

**Table 1:** Prevalence of *A. phagocytophilum* in small mammal species

	Species	Counties	Positive/Total
Dormouse	*Glis glis*	CV	0/43
	***Muscardinus avellanarius***	CJ, **CV**, MS	**1/32**
Hamster	*Cricetus cricetus*	CJ, MS	0/3
Mole	*Talpa europaea*	BH, BZ, CJ, CV, MS, SJ, TL	0/14
Mole-rat	*Spalax leucodon*	TL	0/3
Mouse	*Apodemus agrarius*	BC, CJ, CT, CV, MS	0/60
	***Apodemus flavicollis***	BC, **CJ**, HR, **MS**, **TL**,	**5/77**
	***Apodemus sylvaticus***	CJ, **CT**, CV, HR, MS, **TL**	**2/51**
	***Apodemus uralensis***	**CT**, HR, MS, TL	**3/62**
	*Micromys minutus*	CJ, CT, TL	0/5
	*Mus musculus*	AB, BH, CJ, CV, HR, TL	0/55
	***Mus spicilegus***	BC, CJ, **CT**, TL	**1/50**
Muskrat	*Ondatra zibethicus*	BV, TL	0/9
Rat	*Rattus norvegicus*	AB, CJ, CT, HR, MS	0/10
Shrew	*Crocidura leucodon*	CJ, CT, MS, TL	0/21
	***Crocidura suaveolens***	CJ, CT, CV, MS, **TL**	**1/41**
	*Neomys anomalus*	CT, TL	0/3
	*Neomys fodiens*	CT, MS	0/2
	*Sorex alpinus*	BH	0/1
	***Sorex araneus***	AG, CJ, CV, **HR**, **MS**	**3/33**
	***Sorex minutus***	CJ, CT, CV, HR, MS, **TL**	**1/26**
Squirrel	*Sciurus vulgaris*	BV	0/1
	***Spermophilus citellus***	BT, CT, **TL**	**1/14**
Vole	*Arvicola amphibius*	CT	0/1
	*Arvicola scherman*	BH	0/1
	***Microtus agrestis***	CJ, HR, **TL**	**1/26**
	***Microtus arvalis***	BV, CJ, **CT**, CV, MS, TL	**1/57**
	*Microtus subterraneus*	CJ, HR, MS	0/36
	*Microtus tatricus*	HR	0/1
	*Myodes glareolus*	CJ, CV, HR, MS	0/53

Bold indicates positive samples, hosts and counties with positive animals detected

*Abbreviations*: *AB* Alba, *AG* Argeş, *BC* Bacău, *BT* Botoşani, *BH* Bihor, *BV* Brașov, *BZ* Buzău, *CJ* Cluj, *CT* Constanţa, *CV* Covasna, *HR* Harghita, *MS* Mureş, *SJ* Sălaj, *TL* Tulcea

The positive small mammals originated from Tulcea (6/118; 4.84%, 95% CI: 1.80–10.23%), Constanța (6/153; 3.77%; 95% CI: 1.40–8.03%), Mureş (4/111; 3.48%; 95% CI: 0.96–8.67%), Cluj (2/217; 0.92%; 95% CI: 0.11–3.29%), Harghita (1/27; 3.57%; 95% CI: 0.09–18.35%) and Covasna (1/124; 0.8%; 95% CI: 0.02–4.38%), without significance difference between the counties.

The presence of *A. phagocytophilum* was confirmed by sequence analysis with all the sequences (*n* = 20) showing 99–100% similarity to strains from dogs in Germany and ticks in Belarus and Russia, respectively (GenBank: JX173651, HQ629911, HQ629915). The sequence analysis has shown a small degree of variability with only one up to three nucleotides different between the strains (Additional file 1).

## Discussion

The aim of present study was to evaluate the host spectrum and prevalence of *A. phagocytophilum* in small mammal species across Romania. The results have shown an overall low prevalence, with no significant difference between host species and geographical areas. The low genetic diversity and the large number of positive species from our study confirm the low host specificity of *A. phagocytophilum* observed at least for some variants [13].

*Anaplasma phagocytophilum* DNA was previously detected in several small mammal species such as *A. agrarius*, *A. flavicollis*, *A. uralensis*, *A. sylvaticus*, *M. musculus*, *M. agrestis*, *M. arvalis*, *M. oeconomus*, *Myodes glareolus*, *S. araneus* and *C. russula* in several European countries [8, 13–29]. There are also several reports on the occurrence of *A. phagocytophilum* infection in *Erinaceus europaeus* [30–32], *E. roumanicus* [33] and *Rattus rattus* [15] and in large rodents such as *Hystrix cristata* [34]. In addition to small mammals, *A. phagocytophilum* was detected in a large variety of hosts including birds, domestic and wild carnivores, livestock, wild ruminants, wild boars and humans (reviewed in [13]). To our knowledge, this is the first report of *A. phagocytophilum* in *S. minutus*, *C*. *suaveolens*, *M. spicilegus*, *M. avellanarius* and *S. citellus*.

The overall prevalence in small mammals in the present study was within the prevalence intervals from other reports (reviewed in [13]). The *A. phagocytophilum* prevalence recorded in other European countries present a high variability especially for several species such as: *A. agrarius* (1.28–13.54%) [24, 35], *A. flavicollis* (0.48–18%) [22, 36], *A. sylvaticus* (0.61–11.1%) [19, 20], *M. agrestis* (0.28–25%) [37, 38], *M. arvalis* (0.28–25%) [29, 37] and *M. glareolus* (0.30–21.85%) [25, 28]. In the present study, prevalence of *A. phagocytophilum* in *A. flavicollis* (6.49%) was lower than in the Czech Republic [16] and higher than in Germany and Switzerland [8, 22]. *Apodemus sylvaticus* from Romania was less infected than *A. flavicollis*, in contrast with results obtained in Switzerland [8]. *Anaplasma phagocytophilum* was previously found in one *A. uralensis* in Slovakia, having a similar prevalence (5.6%) with that obtained in our study [23]. The *A. phagocytophilum* prevalence found in *M. agrestis, M. arvalis* and *S. araneus* was lower than in Germany, the UK and Switzerland [8, 23, 39]. A higher prevalence in *S. araneus* than in *Apodemus* spp. was observed in our study, similar with the results from the UK and Switzerland [8, 40]. Our results have shown no *A. phagocytophilum* in *M. glareolus*, while in the majority of studies the bank vole is frequently more infected compared with rodents [8, 23, 26, 36]. Furthermore, in the studies focused on the bank vole, the obtained prevalence ranged between 5 and 22% [14, 28]. The differences in prevalence between different studies could be explained by several factors such as abundance and population structure of the tick vector and the abundance and diversity of potential reservoir hosts, both being influenced by the climatic and ecological features including sampling period. Others factors which may influence the prevalence are related to the type and quality of samples and methods used. Conservative strategies are usually used for screening based on *rrs* and *groEL* genes or multicopy of the major surface proteins such as msp2 and msp4 (reviewed in [41]). Among different types of samples, our previous research and literature data suggest spleen tissue as the most suitable organ for the detection of *A. phagocytophilum* [41].

Small mammals were previously considered reservoir hosts for *A. phagocytophilum* [8]. They are important hosts for immature stages of *I. ricinus* [42], and also for *I. trianguliceps* [38] and *I. hexagonus* [30], which may transmit the infection. However, since most rodents are short-lived animals and that the infection with *A. phagocytophilum* seems to be transient [30], their role as suitable reservoir hosts is currently under debate. Moreover, in recent years it has been suggested that small mammals and their ticks are involved in a separate enzootic cycle. This hypothesis is sustained by the phylogenetic analysis on *ankA* and *groEL* genes which showed that strains isolated from small mammals differ genetically from those circulating in *I. ricinus* ticks, domestic ruminants, wild boar, dogs, horses or humans [26, 28, 39, 43]. It has been suggested that *I. trianguliceps* might be the vector of these rodent strains in the UK [39, 40]. Furthermore, in Switzerland and Slovakia, *A. phagocytophilum* was not detected in *I. ricinus* ticks feeding on rodents even though *A. phagocytophilum* was detected in questing *I. ricinus* in the same areas [44] or in *I. trianguliceps* removed from the same hosts [26]. Moreover, none of the artificially-fed *I. ricinus* became infected after engorging blood from positive rodents [44]. However, more experimental studies, including xenodiagnostics are needed to confirm this separate enzootic cycle. Although the sequence analysis confirmed the presence of *A. phagocytophilum* DNA in small mammal species, experimental studies are required to demonstrate the role of these species as competent host. Moreover, the *rrs* gene is highly conservative and further research on other genes or by more sensitive techniques such as multilocus sequence typing (MLST) or multilocus variable-number tandem-repeat analysis (MLVA), is needed in order to properly characterize these strains present in small mammals in Romania. Based on the data obtained in this study it is unclear if these strains are hazardous to humans or domestic animals and if they are transmitted by public health relevant tick species.

## Conclusions

Several small mammal species are described in the literature as carriers of *A. phagocytophilum.* Besides these, our results have shown other species such as *S. minutus*, *C. suaveolens*, *M. spicilegus*, *M. avellanarius* and *S. citellus*, which can also be infected with *A. phagocytophilum*. However, it is unclear whether these animals can act as competent reservoir hosts and for which *A. phagocytophilum* variants.

## Additional files

Additional file 1: Alignment of the sequences obtained in this study. Nucleotides different between the strains are marked with different colors. (DOCX 19 kb)

## Abbreviations

BLAST: Basic Local Alignments Tool
MLST: Multilocus sequence typing
MLVA: Multilocus variable-number tandem-repeat analysis
nPCR: Nested PCR
*rrs*: *16S* rRNA
