# Supplementary material for: New records for Anaplasma phagocytophilum infection in small mammal species
Source: Parasit Vectors. 2018 Mar 20;11:193. doi: 10.1186/s13071-018-2791-y (PMC5859413; doi:10.1186/s13071-018-2791-y)
Supplement: Supplementary file 1 — Alignment of the sequences obtained in this study. Nucleotides different between the strains are marked with different colors. (DOCX 19 kb) [file 13071_2018_2791_MOESM1_ESM.docx]

1 10 20 30 40 50 60

| | | | | | |

apod flav/bb549 CCGCCTACATGCCCTTTACGCCCAATAATTCCGAACAACGCTTGCCCCCTCCGTATTACC

apod flav/bb726 -CGCCTACATGCCCTTTACGCCCAATAATTCCGAACAACGCTTGCCCCCTCCGTATTACC

apod flav/bb1231 CCGCCTACATGCCCTTTACGCCCAATAATTCCGAACAACGCTTGCCCCCTCCGTATTACC

apod flav/bb1338 CCGCCTACATGCCCTTTACGCCCAATAATTCCGAACAACGCTTGCCCCCTCCGTATTACC

apod flav/cj2225 --GCCTACATGCCCTTTACGCCCAATAATTCCGAACAACGCTTGCCCCCTCCGTATTACC

apod sylv/bb351 --GCCTACATGCCCTTTACGCCCAATAATTCCGAACAACGCTTGCCCCCTCCGTATTACC

apod sylv/cj2220 --GCCTACATGCCCTTTACGCCCAATAATTCCGAACAACGCTTGCCCCCTCCGTATTACC

apod ural/bb1219 -------CATGCCCTTTACGCCCAATAATTCCGAACAACGCTTGCCCCCTCCGTATTACC

apod ural/bb1218 ------ACATGCCCTTTACGCCCAATAATTCCGAACAACGCTTGCCCCCTCCGTATTACC

apod ural/cj194 ---------TGCCCTTTACGCCCAATAATTCCGAACAACGCTTGCCCCCTCCGTATTACC

crocid sauv/cj2351 -----TACATGCCCTTTACGCCCAATAATTCCGAACAACGATTGCCCCCTCCGTATTACC

microt agr/bb359 ---------TGCCCTTTACGCCCAATAATTCCGAACAACGCTTGCCCCCTCCGTATTACC

microt arv/cj4209 ---------TGCCCTTTACGCCCAATAATTCCGAACAACGCTTGCCCCCTCCGTATTACC

mus spicil/cj183 -----------------ACGCCCAATAATTCCGAACAACGCTTGCCCCCTCCGTATTACC

muscard avell/cj2918 -----TACATGCCCTTTACGCCCAATAATTCCGAACAACGCTTGCCCCCTCCGTATTACC

sperm cit/bb634 ---CCTACATGCCCTTTACGCCCAATAATTCCGAACAACGCTTGCCCCCTCCGTATTACC

sorex aran/bb342 --GCCTACATGCCCTTTACGCCCAATAATTCCGAACAACGCTTGCCCCCTCCGTATTACC

sorex aran/bb733 -----TACATGCCCTTTACGCCCAATAATTCCGAACAACGCTTGCCCCCTCCGTATTACC

sorex aran/bb739 --GCCTACATGCCCTTTACGCCCAATAATTCCGAACAACGCTTGCCCCCTCCGTATTACC

sorex min/cj359 ---------TGCCCTTTACGCCCAATAATTCCGAACAACGCTTGCCCCCTCCGTATTACC

apod flav/bb549 GCGGCTGCTGGCACGGAGTTTGCCGGGACTTCTTCTGTAGGTACCGTCATTATCTTCCCT

apod flav/bb726 GCGGCTGCTGGCACGGAGTTTGCCGGGACTTCTTCTGTAGGTACCGTCATTATCTTCCCT

apod flav/bb1231 GCGGCTGCTGGCACGGAGTTTGCCGGGACTTCTTCTGTAGGTACCGTCATTATCTTCCCT

apod flav/bb1338 GCGGCTGCTGGCACGGAGTTTGCCGGGACTTCTTCTGTAGGTACCGTCATTATCTTCCCT

apod flav/cj2225 GCGGCTGCTGGCACGGAGTTTGCCGGGACTTCTTCTGTAGGTACCGTCATTATCTTCCCT

apod sylv/bb351 GCGGCTGCTGGCACGGAGTTTGCCGGGACTTCTTCTGTAGGTACCGTCATTATCTTCCCT

apod sylv/cj2220 GCGGCTGCTGGCACGGAGTTTGCCGGGACTTCTTCTGTAGGTACCGTCATTATCTTCCCT

apod ural/bb1219 GCGGCTGCTGGCACGGAGTTTGCCGGGACTTCTTCTGTAGGTACCGTCATTATCTTCCCT

apod ural/bb1218 GCGGCTGCTGGCACGGAGTTTGCCGGGACTTCTTCTGTAGGTACCGTCATTATCTTCCCT

apod ural/cj194 GCGGCTGCTGGCACGGAGTTTGCCGGGACTTCTTCTGTAGGTACCGTCATTATCTTCCCT

crocid sauv/cj2351 GCGGCTGCTGGCACGGAGTTTGCCGGGACTTCTTCTGTAGGTACCGTCATTATCTTCCCT

microt agr/bb359 GCGGCTGCTGGCACGGAGTTTGCCGGGACTTCTTCTGTAGGTACCGTCATTATCTTCCCT

microt arv/cj4209 GCGGCTGCTGGCACGGAGTTTGCCGGGACTTCTTCTGTAGGTACCGTCATTATCTTCCCT

mus spicil/cj183 GCGGCTGCTGGCACGGAGTTTGCCGGGACTTCTTCTGTAGGTACCGTCATTATCTTCCCT

muscard avell/cj2918 GCGGCTGCTGGCACGGAGTTTGCCGGGACTTCTTCTGTAGGTACCGTCATTATCTTCCCT

sperm cit/bb634 GCGGCTGCTGGCACGGAGTTTGCCGGGACTTCTTCTGTAGGTACCGTCATTATCTTCCCT

sorex aran/bb342 GCGGCTGCTGGCACGGAGTTTGCCGGGACTTCTTCTGTAGGTACCGTCATTATCTTCCCT

sorex aran/bb733 GCGGCTGCTGGCACGGAGTTTGCCGGGACTTCTTCTGTAGGTACCGTCATTATCTTCCCT

sorex aran/bb739 GCGGCTGCTGGCACGGAGTTTGCCGGGACTTCTTCTGTAGGTACCGTCATTATCTTCCCT

sorex min/cj359 GCGGCTGCTGGCACGGAGTTTGCCGGGACTTCTTCTGTAGGTACCGTCATTATCTTCCCT

apod flav/bb549 ACTGAAAGAGTTTTACAACCCTAAGGCCTTCCTCACTCATGCGGCATAGCTGGATCAGGC

apod flav/bb726 ACTGAAAGAGTTTTACAACCCTAAGGCCTTCCTCACTCATGCGGCATAGCTGGATCAGGC

apod flav/bb1231 ACTGAAAGAGTTTTACAACCCTAAGGCCTTCCTCACTCATGCGGCATAGCTGGATCAGGC

apod flav/bb1338 ACTGAAAGAGTTTTACAACCCTAAGGCCTTCCTCACTCATGCGGCATAGCTGGATCAGGC

apod flav/cj2225 ACTGAAAGAGTTTTACAACCCTAAGGCCTTCCTCACTCATGCGGCATAGCTGGATCAGGC

apod sylv/bb351 ACTGAAAGAGTTTTACAACCCTAAGGCCTTCCTCACTCATGCGGCATAGCTGGATCAGGC

apod sylv/cj2220 ACTGAAAGAGTTTTACAACCCTAAGGCCTTCCTCACTCATGCGGCATAGCTGGATCAGGC

apod ural/bb1219 ACTGAAAGAGTTTTACAACCCTAAGGCCTTCCTCACTCATGCGGCATAGCTGGATCAGGC

apod ural/bb1218 ACTGAAAGAGTTTTACAACCCTAAGGCCTTCCTCACTCATGCGGCATAGCTGGATCAGGC

apod ural/cj194 ACTGAAAGAGTTTTACAACCCTAAGGCCTTCCTCACTCATGCGGCATAGCTGGATCAGGC

crocid sauv/cj2351 ACTGAAAGAGTTTTACAACCCTAAGGCCTTCCTCACTCATGCGGCATAGCTGGATCAGGC

microt agr/bb359 ACTGAAAGAGTTTTACAACCCTAAGGCCTTCCTCACTCATGCGGCATAGCTGGATCAGGC

microt arv/cj4209 ACTGAAAGAGTTTTACAACCCTAAGGCCTTCCTCACTCACGCGGCATAGCTGGATCAGGC

mus spicil/cj183 ACTGAAAGAGTTTTACAACCCTAAGGCCTTCCTCACTCATGCGGCATAGCTGGATCAGGC

muscard avell/cj2918 ACTGAAAGAGTTTTACAACCCTAAGGCCTTCCTCACTCATGCGGCATAGCTGGATCAGGC

sperm cit/bb634 ACTGAAAGAGTTTTACAACCCTAAGGCCTTCCTCACTCATGCGGCATAGCTGGATCAGGC

sorex aran/bb342 ACTGAAAGAGTTTTACAACCCTAAGGCCTTCCTCACTCATGCGGCATAGCTGGATCAGGC

sorex aran/bb733 ACTGAAAGAGTTTTACAACCCTAAGGCCTTCCTCACTCATGCGGCATAGCTGGATCAGGC

sorex aran/bb739 ACTGAAAGAGTTTTACAACCCTAAGGCCTTCCTCACTCATGCGGCATAGCTGGATCAGGC

sorex min/cj359 ACTGAAAGAGTTTTACAACCCTAAGGCCTTCCTCACTCATGCGGCATAGCTGGATCAGGC

apod flav/bb549 TTGCGCCCATTGTCCAATATTCCCCACTGCTGCCTCCCGTAGGAGTCTGAACCGTATCTC

apod flav/bb726 TTGCGCCCATTGTCCAATATTCCCCACTGCTGCCTCCCGTAGGAGTCTGAACCGTATCTC

apod flav/bb1231 TTGCGCCCATTGTCCAATATTCCCCACTGCTGCCTCCCGTAGGAGTCTGAACCGTATCTC

apod flav/bb1338 TTGCGCCCATTGTCCAATATTCCCCACTGCTGCCTCCCGTAGGAGTCTGAACCGTATCTC

apod flav/cj2225 TTGCGCCCATTGTCCAATATTCCCCACTGCTGCCTCCCGTAGGAGTCTGAACCGTATCTC

apod sylv/bb351 TTGCGCCCATTGTCCAATATTCCCCACTGCTGCCTCCCGTAGGAGTCTGGACCGTATCTC

apod sylv/cj2220 TTGCGCCCATTGTCCAATATTCCCCACTGCTGCCTCCCGTAGGAGTCTGGACCGTATCTC

apod ural/bb1219 TTGCGCCCATTGTCCAATATTCCCCACTGCTGCCTCCCGTAGGAGTCTGGACCGTATCTC

apod ural/bb1218 TTGCGCCCATTGTCCAATATTCCCCACTGCTGCCTCCCGTAGGAGTCTGGACCGTATCTC

apod ural/cj194 TTGCGCCCATTGTCCAATATTCCCCACTGCTGCCTCCCGTAGGAGTCTGGACCGTATCTC

crocid sauv/cj2351 TTGCGCCCATTGTCCAATATTCCCCACTGCTGCCTCCCGTAGGAGTCTGGACCGTATCTC

microt agr/bb359 TTGCGCCCATTGTCCAATATTCCCCACTGCTGCCTCCCGTAGGAGTCTGGACCGTATCTC

microt arv/cj4209 TTGCGCCCATTGTCCAATATTCCCCACTGCTGCCTCCCGTAGGAGTCTGGACCGTATCTC

mus spicil/cj183 TTGCGCCCATTGTCCAATATTCCCCACTGCTGTCTCCCGTAGGAGTCTGGACCGTATCTC

muscard avell/cj2918 TTGCGCCCATTGTCCAATATTCCCCACTGCTGCCTCCCGTAGGAGTCTGGACCGTATCTC

sperm cit/bb634 TTGCGCCCATTGTCCAATATTCCCCACTGCTGCCTCCCGTAGGAGTCTGGACCGTATCTC

sorex aran/bb342 TTGCGCCCATTGTCCAATATTCCCCACTGCTGCCTCCCGTAGGAGTCTGGACCGTATCTC

sorex aran/bb733 TTGCGCCCATTGTCCAATATTCCCCACTGCTGCCTCCCGTAGGAGTCTGGACCGTATCTC

sorex aran/bb739 TTGCGCCCATTGTCCAATATTCCCCACTGCTGCCTCCCGTAGGAGTCTGGACCGTATCTC

sorex min/cj359 TTGCGCCCATTGTCCAATATTCCCCACTGCTGCCTCCCGTAGGAGTCTGGACCGTATCTC

apod flav/bb549 AGTTCCAGTGTGGCTGATCATCCTCTCAGACCAGCTATAGATCATCGCCTTGGTAGGCCT

apod flav/bb726 AGTTCCAGTGTGGCTGATCATCCTCTCAGACCAGCTATAGATCATCGCCTTGGTAGGCCT

apod flav/bb1231 AGTTCCAGTGTGGCTGATCATCCTCTCAGACCAGCTATAGATCATCGCCTTGGTAGGCCT

apod flav/bb1338 AGTTCCAGTGTGGCTGATCATCCTCTCAGACCAGCTATAGATCATCGCCTTGGTAGGCCT

apod flav/cj2225 AGTTCCAGTGTGGCTGATCATCCTCTCAGACCAGCTATAGATCATCGCCTTGGTAGGCCT

apod sylv/bb351 AGTTCCAGTGTGGCTGATCATCCTCTCAGACCAGCTATAGATCATCGCCTTGGTAGGCCT

apod sylv/cj2220 AGTTCCAGTGTGGCTGATCATCCTCTCAGACCAGCTATAGATCATCGCCTTGGTAGGCCT

apod ural/bb1219 AGTTCCAGTGTGGCTGATCATCCTCTCAGACCAGCTATAGATCATCGCCTTGGTAGGCCT

apod ural/bb1218 AGTTCCAGTGTGGCTGATCATCCTCTCAGACCAGCTATAGATCATCGCCTTGGTAGGCCT

apod ural/cj194 AGTTCCAGTGTGGCTGATCATCCTCTCAGACCAGCTATAGATCATCGCCTTGGTAGGCCT

crocid sauv/cj2351 AGTTCCAGTGTGGCTGATCATCCTCTCAGACCAGCTATAGATCATCGCCTTGGTAGGCCT

microt agr/bb359 AGCTCCAGTGTGGCTGATCATCCTCTCAGACCAGCTATAGATCATCGCCTTGGTAGGCCT

microt arv/cj4209 AGTTCCAGTGTGGCTGATCATCCTCTCAGACCAGCTATAGATCATCGCCTTGGTAGGCCT

mus spicil/cj183 AGTTCCAGTGTGGCTGATCATCCTCTCAGACCAGCTATAGATCATCGCCTTGGTAGGCCT

muscard avell/cj2918 AGTTCCAGTGTGGCTGATCATCCTCTCAGACCAGCTATAGATCATCGCCTTGGTAGGCCT

sperm cit/bb634 AGTTCCAGTGTGGCTGATCATCCTCTCAGACCAGCTATAGATCATCGCCTTGGTAGGCCT

sorex aran/bb342 AGTTCCAGTGTGGCTGATCATCCTCTCAGACCAGCTATAGATCATCGCCTTGGTAGGCCT

sorex aran/bb733 AGTTCCAGTGTGGCTGATCATCCTCTCAGACCAGCTATAGATCATCGCCTTGGTAGGCCT

sorex aran/bb739 AGTTCCAGTGTGGCTGATCATCCTCTCAGACCAGCTATAGATCATCGCCTTGGTAGGCCT

sorex min/cj359 AGTTCCAGTGTGGCTGATCATCCTCTCAGACCAGCTATAGATCATCGCCTTGGTAGGCCT

apod flav/bb549 TTACCCTACCAACTAGCTAATCTAACATAGGCTCATCTAATAGCGATAAATCTTTCCCCC

apod flav/bb726 TTACCCTACCAACTAGCTAATCTAACATAGGCTCATCTAATAGCGATAAATCTTTCCCCC

apod flav/bb1231 TTACCCTACCAACTAGCTAATCTAACATAGGCTCATCTAATAGCGATAAATCTTTCCCCC

apod flav/bb1338 TTACCCTACCAACTAGCTAATCTAACATAGGCTCATCTAATAGCGATAAATCTTTCCCCC

apod flav/cj2225 TTACCCTACCAACTAGCTAATCTAACATAGGCTCATCTAATAGCGATAAATCTTTCCCCC

apod sylv/bb351 TTACCCTACCAACTAGCTAATCTAACATAGGCTCATCTAATAGCGATAAATCTTTCCCCC

apod sylv/cj2220 TTACCCTACCAACTAGCTAATCTAACATAGGCTCATCTAATAGCGATAAATCTTTCCCCC

apod ural/bb1219 TTACCCTACCAACTAGCTAATCTAACATAGGCTCATCTAATAGCGATAAATCTTTCCCCC

apod ural/bb1218 TTACCCTACCAACTAGCTAATCTAACATAGGCTCATCTAATAGCGATAAATCTTTCCCCC

apod ural/cj194 TTACCCTACCAACTAGCTAATCTAACATAGGCTCATCTAATAGCGATAAATCTTTCCCCC

crocid sauv/cj2351 TTACCCTACCAACTAGCTAATCTAACATAGGCTCATCTAATAGCGATAAATCTTT**-**CCCC

microt agr/bb359 TTACCCTACCAACTAGCTAATCTAACATAGGCTCATCTAATAGCGATAAATCTTTCCCCC

microt arv/cj4209 TTACCCTACCAACTAGCTAATCTAACATAGGCTCATCTAATAGCGATAAATCTTTCCCCC

mus spicil/cj183 TTACCCTACCAACTAGCTAATCTAACATAGGCTCATCTAATAGCGATAAATCTTTCCCCC

muscard avell/cj2918 TTACCCTACCAACTAGCTAATCTAACATAGGCTCATCTAATAGCGATAAATCTTTCCCCC

sperm cit/bb634 TTACCCTACCAACTAGCTAATCTAACATAGGCTCATCTAATAGCGATAAATCTTTCCCCC

sorex aran/bb342 TTACCCTACCAACTAGCTAATCTAACATAGGCTCATCTAATAGCGATAAATCTTTCCCCC

sorex aran/bb733 TTACCCTACCAACTAGCTAATCTAACATAGGCTCATCTAATAGCGATAAATCTTTCCCCC

sorex aran/bb739 TTACCCTACCAACTAGCTAATCTAACATAGGCTCATCTAATAGCGATAAATCTTTCCCCC

sorex min/cj359 TTACCCTACCAACTAGCTAATCTAACATAGGCTCATCTAATAGCGATAAATCTTTCCCCC

apod flav/bb549 GCAGGGATTATACAGTATTACCCACCATTTCTAGTGGCTATCCCATACTACTAGGT----

apod flav/bb726 GCAGGGATTATACAGTATTACCCACCATTTCTAGTGGCTATCCCATACTACTAGG-----

apod flav/bb1231 GCAGGGATTATACAGTATTACCCACCATTTCTAGTGGCTATCCCATACTACTAGG-----

apod flav/bb1338 GCAGGGATTATACAGTATTACCCACCATTTCTAGTGGCTATCCCATACTACTAGGT----

apod flav/cj2225 GCAGGGATTATACAGTATTACCCACCATTTCTAGTGGCTATCCCATACTACTAGGT----

apod sylv/bb351 GCAGGGATTATACAGTATTACCCACCATTTCTAGTGGCTATCCCATACTACTAGGTAGAT

apod sylv/cj2220 GCAGGGATTATACAGTATTACCCACCATTTCTAGTGGCTATCCCATACTACTAGGTAGAT

apod ural/bb1219 GCAGGGATTATACAGTATTACCCACCATTTCTAGTGGCTATCCCATACTACTAGGTAGAT

apod ural/bb1218 GCAGGGATTATACAGTATTACCCACCATTTCTAGTGGCTATCCCATACTACTAGGTAGAT

apod ural/cj194 GCAGGGATTATACAGTATTACCCACCATTTCTAGTGGCTATCCCATACTACTAGGTAGAT

crocid sauv/cj2351 GCAGGGATTATACAGTATTACCCACCATTTCTAGTGGCTATCCCATACTACTAGGTAGAT

microt agr/bb359 GCAGGGATTATACAGTATTACCCACCATTTCTAGTGGCTATCCCATACTACTAGGTAGAT

microt arv/cj4209 GCAGGGATTATACAGTATTACCCACCATTTCTAGTGGCTATCCCATACTACTAGGTAGAT

mus spicil/cj183 GCAGGGATTATACAGTATTACCCACCATTTCTAGTGGCTATCCCATACTACTAGGTAGAT

muscard avell/cj2918 GCAGGGATTATACAGTATTACCCACCATTTCTAGTGGCTATCCCATACTACTAGGTAGAT

sperm cit/bb634 GCAGGGATTATACAGTATTACCCACCATTTCTAGTGGCTATCCCATACTACTAGGTAGAT

sorex aran/bb342 GCAGGGATTATACAGTATTACCCACCATTTCTAGTGGCTATCCCATACTACTAGGTAGAT

sorex aran/bb733 GCAGGGATTATACAGTATTACCCACCATTTCTAGTGGCTATCCCATACTACTAGGTAGAT

sorex aran/bb739 GCAGGGATTATACAGTATTACCCACCATTTCTAGTGGCTATCCCATACTACTAGGTAGAT

sorex min/cj359 GCAGGGATTATACAGTATTACCCACCATTTCTAGTGGCTATCCCATACTACTAGGTAGAT

apod flav/bb549 ------------------------------------------------------------

apod flav/bb726 ------------------------------------------------------------

apod flav/bb1231 ------------------------------------------------------------

apod flav/bb1338 ------------------------------------------------------------

apod flav/cj2225 ------------------------------------------------------------

apod sylv/bb351 TCCTATGCATTACTCACCCGTCTGCCACTAATTATTCTTTATAGCAAGCTATAAAG----

apod sylv/cj2220 TCCTATGCATTACTCACCCGTCTGCCACTAATTATTCTTTATAGCAAGCTATAAAG----

apod ural/bb1219 TCCTATGCATTACTCACCCGTCTGCCACTAACTATTCTTTATAGCAAGCTATAAA-----

apod ural/bb1218 TCCTATGCATTACTCACCCGTCTGCCACTAACTATTCTTTATAGCAAGCTATAA------

apod ural/cj194 TCCTATGCATTACTCACCCGTCTGCCACTAACTATTCTTTATAGCAAGCTATAAA-----

crocid sauv/cj2351 TCCTATGCATTACTCACCCGTCTGCCACTAACTATTCTTTATAGCAAGCTATAAA-----

microt agr/bb359 TCCTATGCATTACTCACCCGTCTGCCACTAACTATTCTTTATAGCAAGCTATAAA-----

microt arv/cj4209 TCCTATGCATTACTCACCCGTCTGCCACTAATTGTTCTTTGTAGCAAGCTATAAAG----

mus spicil/cj183 TCCTATGCATTACTCACCCGTCTGCCACTAACTATTCTTTATAGCAAGCTATAAA-----

muscard avell/cj2918 TCCTATGCATTACTCACCCGTCTGCCACTAACTATTCTTTATAGCAAGCTATAAAG----

sperm cit/bb634 TCCTATGCATTACTCACCCGTCTGCCACTAACTATTCTTTATAGCAAGCTATAAAG----

sorex aran/bb342 TCCTATGCATTACTCACCCGTCTGCCACTAACTATTCTTTATAGCAAGCTAGAAA-----

sorex aran/bb733 TCCTATGCATTACTCACCCGTCTGCCACTAACTATTCTTTATAGCAAG------------

sorex aran/bb739 TCCTATGCATTACTCACCCGTCTGCCACTAACTATTCTTTATAGCAAGCTA---------

sorex min/cj359 TCCTATGCATTACTCACCCGTCTGCCACTAATTATTCTTTATAGCAAGCTATAAAGATAA

apod flav/bb549 ------

apod flav/bb726 ------

apod flav/bb1231 ------

apod flav/bb1338 ------

apod flav/cj2225 ------

apod sylv/bb351 ------

apod sylv/cj2220 ------

apod ural/bb1219 ------

apod ural/bb1218 ------

apod ural/cj194 ------

crocid sauv/cj2351 ------

microt agr/bb359 ------

microt arv/cj4209 ------

mus spicil/cj183 ------

muscard avell/cj2918 ------

sperm cit/bb634 ------

sorex aran/bb342 ------

sorex aran/bb733 ------

sorex aran/bb739 ------

sorex min/cj359 ATCCGT
